# Nutritional Status and Oral Frailty: A Community Based Study

**DOI:** 10.3390/nu12092886

**Published:** 2020-09-21

**Authors:** Yoshiaki Nomura, Yoshimasa Ishii, Shunsuke Suzuki, Kenji Morita, Akira Suzuki, Senichi Suzuki, Joji Tanabe, Yasuo Ishiwata, Koji Yamakawa, Yota Chiba, Meu Ishikawa, Kaoru Sogabe, Erika Kakuta, Ayako Okada, Ryoko Otsuka, Nobuhiro Hanada

**Affiliations:** 1 Department of Translational Research, Tsurumi University School of Dental Medicine, Yokohama 230-8501, Japan; ishikawa-me@tsurumi-u.ac.jp (M.I.); sogabe-k@tsurumi-u.ac.jp (K.S.); otsuka-ryoko@tsurumi-u.ac.jp (R.O.); hanada-n@tsurumi-u.ac.jp (N.H.); 2 Ebina Dental Association, Kanagawa 243-0421, Japan; ishiiryo141@gmail.com (Y.I.); shun-s@wg8.so-net.ne.jp (S.S.); morita-d-c-2@t06.itscom.net (K.M.); suzuki@bell-dental.com (A.S.); lion@kd5.so-net.ne.jp (S.S.); tanabedental5@me.com (J.T.); yasuo-i@rb3.so-net.ne.jp (Y.I.); cherry@cherry-dental.com (K.Y.); yota@db3.so-net.ne.jp (Y.C.); 3 Department of Oral Microbiology, Tsurumi University School of Dental Medicine, Yokohama 230-8501, Japan; kakuta-erika@tsurumi-u.ac.jp; 4 Department of Operative Dentistry, Tsurumi University School of Dental Medicine, Yokohama 230-8501, Japan; okada-a@tsurumi-u.ac.jp

**Keywords:** nutritional status, population survey, oral frailty, health behavior

## Abstract

Compromised oral health can alter food choices. Poor masticatory function leads to imbalanced food intake and undesirable nutritional status. The associations among nutritional status, oral health behavior, and self-assessed oral functions status were investigated using a community-based survey. In total, 701 subjects more than 50 years old living Ebina city located southwest of the capital Tokyo were investigated. The number of remaining teeth was counted by dental hygienists. Oral health behavior and self-assessed oral functions were evaluated by oral frailty checklist. Nutritional status was evaluated by the brief-type self-administered diet history questionnaire using Dietary Reference Intakes for Japanese as reference. More than 80% of subjects’ intakes of vitamin B_12_, pantothenic acid, copper, and proteins were sufficient. In contrast, only 19% of subjects’ intake of vitamin A was sufficient and 35.5% for vitamin B_1_. More than 90% of subjects’ intakes of vitamin D and vitamin K were sufficient. Only 35.5% of subjects’ intakes of dietary fiber were sufficient. Overall, 88.9% of subjects had excess salt. The number of remaining teeth was not correlated with nutritional intakes. Oral health behavior significantly correlated with nutritional intakes. Oral functions are important for food choice; however, oral functions were not directly correlated with nutritional intakes. Comprehensive health instructions including nutrition and oral health education is necessary for health promotion.

## 1. Introduction

Compromised oral health status can alter food choices, leading to suboptimal nutritional status. The associations among dietary practices, nutritional status, and oral health status are complex with many interrelated factors. 

Oral health status is associated with various diseases and quality of life. Two main pathways have been suggested for the relationship between oral health status and general health: odontogenic bacteremia [1,2] and malnutrition by deteriorated masticatory function [3,4,5]. Mastication is an important function of the oral cavity. Masticatory function is strongly suggested to be associated with general health. Decreased masticatory function changes food preferences and disturbs balance of food intake [6,7]. In older persons, the number of teeth affects the intake of some foods and nutrients [8,9]. Poor masticatory function leads to imbalanced food intake and undesirable nutritional status that can lead to chronic systemic illness. Especially in older persons, a relationship between masticatory function and mortality has been suggested [10,11].

Oral frailty is defined as a mild decline in oral function, and it is reversible in the early stage. Therefore, early detection and treatment of oral frailty is very useful. Prevention of oral frailty can be expected to reduce medical and nursing care cost. Fewer than 20 remaining teeth, articulatory oral motor skill, weak tongue pressure, difficulties eating tough foods, and difficulties in swallowing tea or soup are risk factors for physical frailty, sarcopenia, and disability [12]. Therefore, the concept of oral frailty was widely introduced in Japan [12,13]. 

Fermentable carbohydrate, especially sugar, causes dental caries, and thus the relationship between sugar intake and dental caries has been intensively studied. Dental researchers were involved as guideline editors for sugar intake published by World Health Organization [14]. However, studies on the relationship among oral health status, nutritional status, and nutrient intake other than sugar consumption are still insufficient [15,16]. Poor dietary intake increased the risk of periodontal disease. Inverse associations were found between fatty acids, vitamin C, vitamin E, beta-carotene, fiber, calcium, dairy, fruits, and vegetables and risk of periodontal disease [17]. The relationship between oral health and nutrition is primarily related to masticatory function, but it is complicated by other factors such as race, culture, lifestyle, and personal preferences [18,19].

The basic health policy of the Japanese government is to prevent the onset and aggravation of major noncommunicable diseases as well as frailty in older persons. Insufficient or imbalanced nutritional intake is one of the major common risk factors for the noncommunicable diseases and frailty. “Dietary Reference Intakes for Japanese” was formulated and has been revised every five years under the Health Promotion Act [20,21]. Target values have been set for the purpose of maintaining/promoting health, preventing the onset and progression of noncommunicable diseases, and prevention of malnutrition and frailty in the elderly. The target values for each of the nutrients are set according to the gender and age groups. Meeting these values is desirable to prevent non-communicable diseases and frailty. 

For the assessment of nutritional intake for Japanese people, a validated questionnaire has been developed and named as the brief-type self-administered diet history questionnaire (BDHQ) [22,23,24,25]. BDHQ has been generally used in epidemiological studies carried out in Japan from elementary school children to older Japanese [22,23,24,25].

Few studies have investigated the relationship between oral functions and nutritional status [26]. In this study, the number of remaining teeth was recorded, while self-assessed oral functions and nutritional intake on a community basis were investigated using BDHQ and evaluated using the Dietary Reference Intakes for Japanese, respectively. The purpose of this study was to examine the impact of oral frailty on nutrient intake levels and nutrient intake at a community level.

## 2. Materials and Methods

### 2.1. Setting

A questionnaire on oral frail was distributed for the citizens of Ebina city, located near the capital of Tokyo. A booth was set for the survey outside of city hall, a housing estate, and a sports center from December 2018 to January 2019. Before distribution, age was asked. The questionnaire was distributed to subjects more than 50 years old. In the booth, dental hygienists counted the number of remaining teeth under the supervision of dentists. We recommended all subjects visit a dental office. Fifty-five subjects (7.8%) agreeing to visit a dental office were invited to a dental office and the number of remaining teeth were again counted. The representativeness of the participants in this study was confirmed by comparing three major nutrient intakes and self-reported number of remaining teeth obtained from the Japanese national health and nutrition examination survey conducted at 2018 [27]. 

### 2.2. Questionnaire

The oral frailty checklist proposed by the Japan dental association was used. The checklist consisted by 8 items: (1) harder to eat hard food than half a year ago (difficult to eat hard food); (2) sometimes choke on tea or soup (choking); (3) do you use dentures (using denture); (4) minding about oral dryness (Xerostomia); (5) less frequently going out than half a year ago (less frequently going out); (6) capable of chewing hard food such as pickled radish or shredded and dried squid (feasible to chew hard food); (7) brushing teeth at least twice a day (brushing teeth at least twice a day); and (8) attending dentist at least once a year (regular attendance of dental clinic).

By the standard protocol, if subjects answered yes to Item 1, 2 or 3, two points were given for each answer. If subjects answered yes to Item 4, 5 or 6, one point was given for each answer. If subjects answered no to Item 7, 8 or 9, one point was given for each answer. The maximum score was 11. The screening criterion was defined by the sum of the scores: Low risk for 0–2 points, moderate risk for 3 points, and high risk for more than 4 points. 

### 2.3. Dietary Reference Intakes for Japanese (2015)

Japanese Minister of Health, Labor and Welfare formulated Dietary Reference Intakes for Japanese are in accordance with Article 30-2 of the Health Promotion Act (Act No. 103 of 2002) [20,21]. It proposes target values of desirable dietary intake of energy and nutrients for Japanese people to maintain and promote their health. Dietary reference intakes (DRIs) were determined based on scientific findings where data were available. It determined reference values for 34 nutrients and energy intakes.

For nutrients, five reference values were determined: Estimated Average Requirement (EAR), Recommended Dietary Allowance (RDA), Adequate Intake (AI), Tolerable Upper Intake Level (UL), and Tentative Dietary Goal for preventing LRDs (DG). 

The EARs indicate the amount that would meet the nutrient requirements of 50% of the population. The RDA indicates the amount that would meet the requirement of most of the population. The Adequate Intake (AI) was developed where EAR and RDA could not be set due to insufficient scientific evidence. For the purpose of avoiding adverse health effects due to excessive intake, Tolerable Upper Intake Level (UL) was determined. For the purpose of prevention of Lifestyle-Related Diseases (LRDs), Tentative Dietary Goal for preventing LRDs (DG) was developed. Reference values were determined separately with respect to gender and age group. 

Target BMI range is presented in this Dietary Reference Intakes for Japanese. Target BMI range is presented by age groups, and it is common to men and women: 20.0–24.9 (kg/m^2^) for 50–69 years old, 21.5–24.9 (kg/m^2^) for 70 years older.

### 2.4. Brief-Type Self-Administered Diet History Questionnaire (BDHQ)

For the evaluation of the nutritional status, the brief-type self-administered diet history questionnaire (BDHQ) was used. The BDHQ asks about the consumption frequency of selected foods to estimate the dietary intake of fifty-eight food and beverage items during the preceding month. Details of the BDHQ’s structure, method of calculating dietary intake are described by its developers [16,17,18,19,20]. Calculation of nutritional intakes was ordered from the DHQ support center (Gender Medical Research, Co. Ltd., Tokyo, Japan).

Calculated nutritional status was classified by the target values by Dietary Reference Intakes for Japanese [20,21].

### 2.5. Examinations

In the survey booth, the number of remaining teeth was counted by a dental hygienist using penlight, disposable dental mirror, and probe. Height and body weight were measured. Body Mass Index (BMI) was calculated by the following formula:

BMI = body weight (kg) ÷ (body height (m))^2^ (1)

### 2.6. Statistical Analysis

Descriptive analysis was performed by SPSS version 24.0 (IBM, Tokyo, Japan). For contentious variables, normality was checked by Kolmogorov–Smirnov test. For the comparisons of groups, t-test, Mann–Whitney U test, or Kruskal–Wallis test was applied by the normality of distribution. To visualize correlations, structural equation modeling (SEM) was carried out by AMOS version 24.0 (IBM, Tokyo, Japan). 

### 2.7. Ethics

Informed written consent was obtained simultaneously at the time of collection of questionnaire. The study protocol was approved by the Ethical Committee of Tsurumi University School of Dental Medicine (approval number: 1747).

## 3. Results

### 3.1. Characteristics of the Participants

The distribution and collection of the questionnaire was conducted simultaneously. Therefore, all questionnaires were collected. The data of 701 subjects (351 men, 350 women, mean age 71.5 ± 8.9) were analyzed. Their numbers and proportion in age groups were: 50–59 years old, 49 (7.0%) for men and 40 (5.7%) for women; 60–69 years old, 99 (14.1%) for men and 97 (13.8%) for women; 70–79 years old, 148 (21.1%) for men and 149 (21.3%) for women; and more than 80 years old, 55 (7.8%) for men and 64 (9.1%) for women.

The results of the representativeness comparing Japanese national health and nutritional survey are shown in Table S1.

### 3.2. Nutritional Status and Body Mass Index

#### 3.2.1. Proportion of Energy Intake and Body Mass Index (BMI)

**Figure 1:**
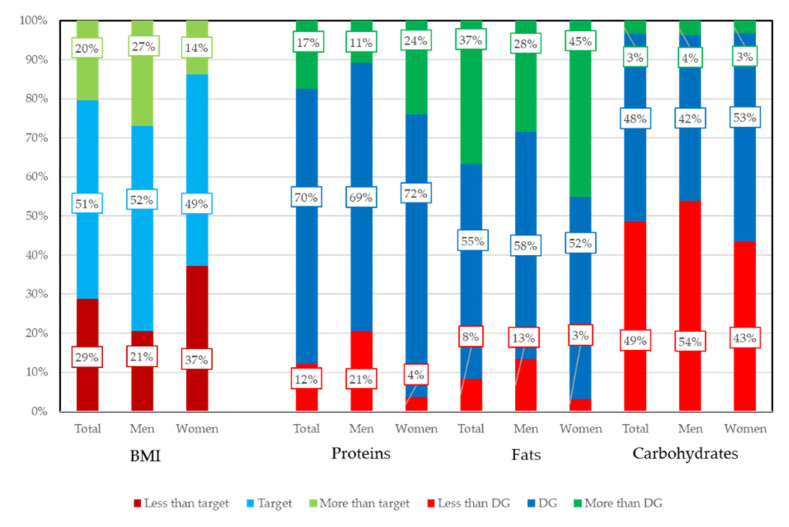
Proportion of subjects for BMI and meeting Tentative Dietary Goal for preventing lifestyle related diseases. Energy balance on three major nutrients are set in Dietary Reference Intakes for Japanese as Tentative Dietary Goal for preventing lifestyle related diseases (DG). DG for three major nutrients are expressed by percent of energy intakes. When comparing men and women, distributions were all statistically significant by χ^2^ tests. Target BMI ranges are 20.0–24.9 (kg/m^2^) for 50–69 years old and 21.5–24.9 (kg/m^2^) for 70 years or older. Target BMI range is common to men and women. Optimal range of Body Mass Index (Target BMI) and Tentative Dietary Goal for preventing lifestyle related diseases (DG) are set in Dietary Reference Intakes for Japanese.

Energy balance on three major nutrients are set in Dietary Reference Intakes for Japanese as Tentative Dietary Goal for preventing lifestyle related diseases (DG). Subjects within DG range were 70.5% for proteins, 54.9% for fats, and 36.8% for carbohydrates. The results are shown in Figure 1. When comparing men and women, the distributions were all statistically significant (BMI, proteins, and fats, *p* < 0.01; carbohydrates, *p* = 0.014). 

The three major nutrients (proteins, fats, and carbohydrates) of DG are set as energy balance by the ratio of energy intake. For proteins, 75% of subjects were in the DG range (13–20%); 36.6% of subjects exceeded the DG range (20–30%) of fats; and 48.6% were less than the DG range (50–65%) for carbohydrates. The proportion of woman with less than target value of BMI was higher than men. Proportion of men with more than target value of BMI was higher than women. The proportion of men whose intakes of macronutrients were less than TG was higher than women. The proportion of women whose intakes of proteins and fats were more than TG was higher than women.

There were 356 subjects within target BMI (50.8%). Scatter plots of BMI against the proportion of energy intakes are shown in Figure S2. Negative correlations were observed for three nutrients against BMI, and the statistically significant correlations were observed between proteins and BMI and between carbohydrate and BMI. However, the correlations were very weak: protein = −0.096 (*p* = 0.011), fats = −0.041(*p* = 0.283), and carbohydrates = −0.138 (*p* < 0.001).

Cross tabulation of BMI and three macronutrients by categorize values by DG and target value is shown in Table 1. *p*-values except for Fats were statistically significant (proteins, *p* = 0.017; fats, *p* = 0.091; carbohydrates, *p* = 0. 002). For continuous variables, *p*-values except for Fats were statistically significant. Differences in the number of remaining teeth, BMI, and the three macronutrients by categorical values of DG are shown in Table S2.

#### 3.2.2. Vitamins, Macro Minerals, and Micro Minerals

**Figure 2:**
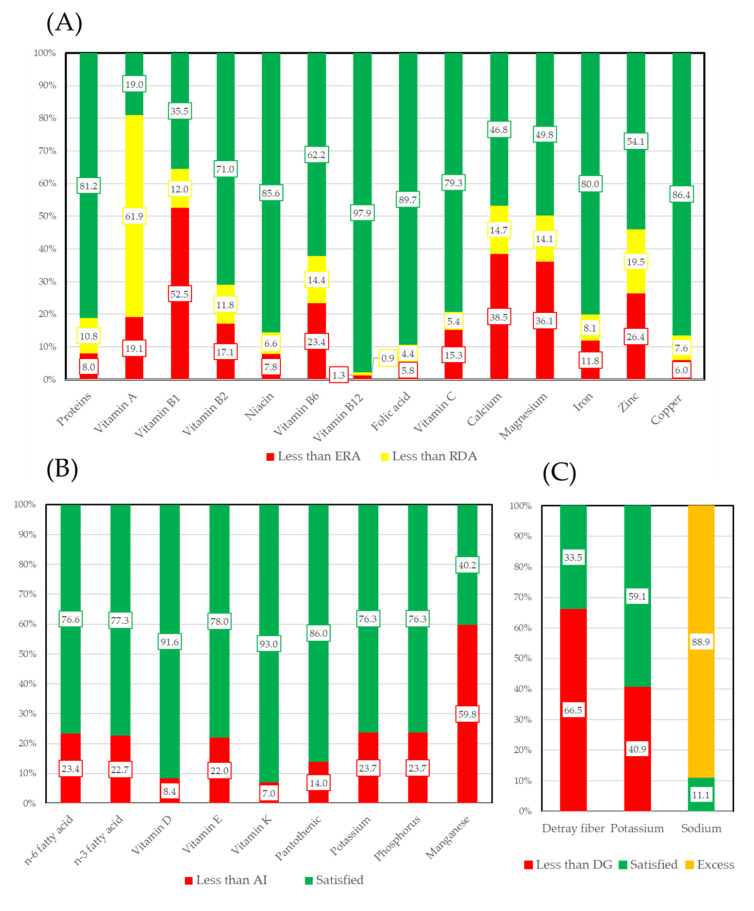
Meeting of vitamins, macro minerals, and micro minerals. Levels were determined by Dietary Reference Intakes for Japanese. Figure 2 shows the proportion of subjects whose intake of each nutrient was sufficient based (**A**) EAR and RDA. (**B**) AI. (**C**) DG. EAR: Estimated Average Requiremen. RDA: Recommended Dietary Allowance. AI: Adequate Intake. DG: Tentative Dietary Goal for preventing LRDs.

The intakes of vitamins, macro minerals, and micro minerals were categorized by EAR (Estimated Average Requirement) and RDA (Recommended Dietary Allowance) or AI (Adequate Intake) according to Dietary Reference Intakes for Japanese (2015). Figure 2 shows the proportion of subjects whose intake of each nutrient was sufficient based on EAR and RDA (Figure 2A) and AI (Figure 2B). Additionally, fiber, which only has a DG set, is also presented. More than 80% of subjects’ intakes of vitamin B_12_, pantothenic acid, copper, and proteins were sufficient. In contrast, only 19% of subjects’ intakes of vitamin A and 35.5% of subjects’ intakes of vitamin B_1_ were sufficient. More than 90% of subjects’ intakes of vitamin D and vitamin K were sufficient. Only 35.5% of subjects’ intakes of dietary fiber were sufficient. Sodium is converted as kitchen salts and a DG set. Overall, 88.9% of subjects had excess salt.

### 3.3. Oral Health Status of the Participants

The number of remaining teeth of the subjects participated in this study was 20.8 ±8.4 for men (median, 24; 25th–75th, 17–27), 21.6 ± 7.6 for women, (median, 24; 25th–75th, 19–27), and 21.22 ± 8.02 for total (median, 24; 25th–75th, 18–27). The difference was not statistically significant by Mann–Whitney U test. The proportion of subjects with more than 20 remaining teeth was 70.0% for men, 73.9% for women, and 72.0% for total.

The results of oral frailty screening questionnaire are summarized in Table 2.

### 3.4. Oral Health Status and Nutritional Status

**Figure 3:**
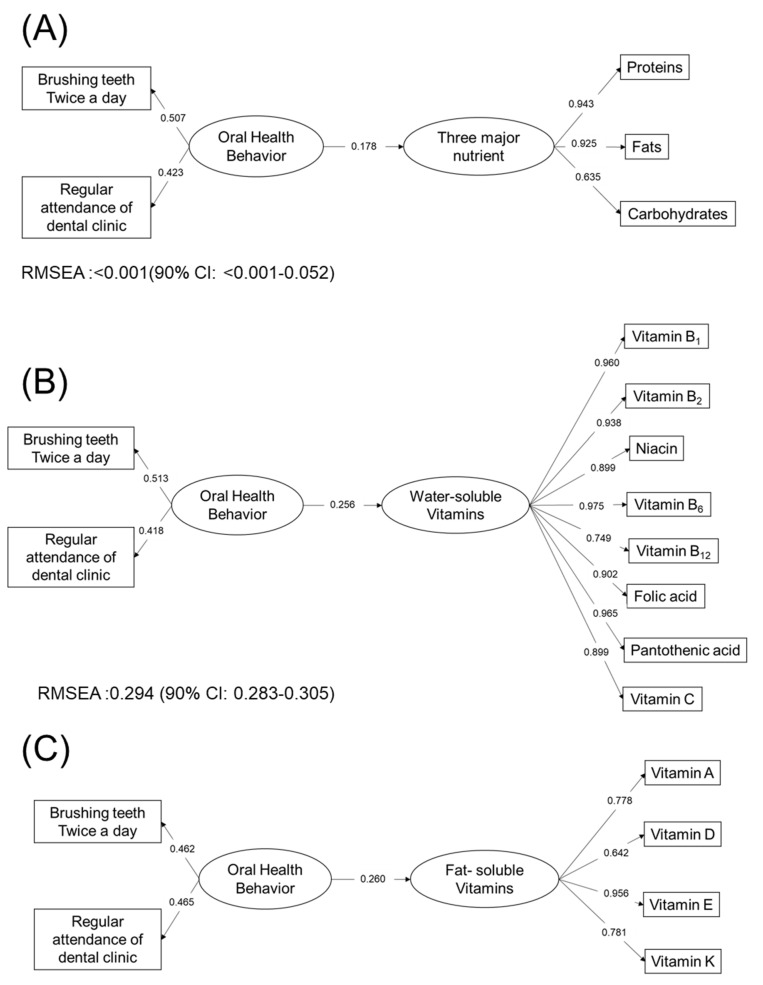

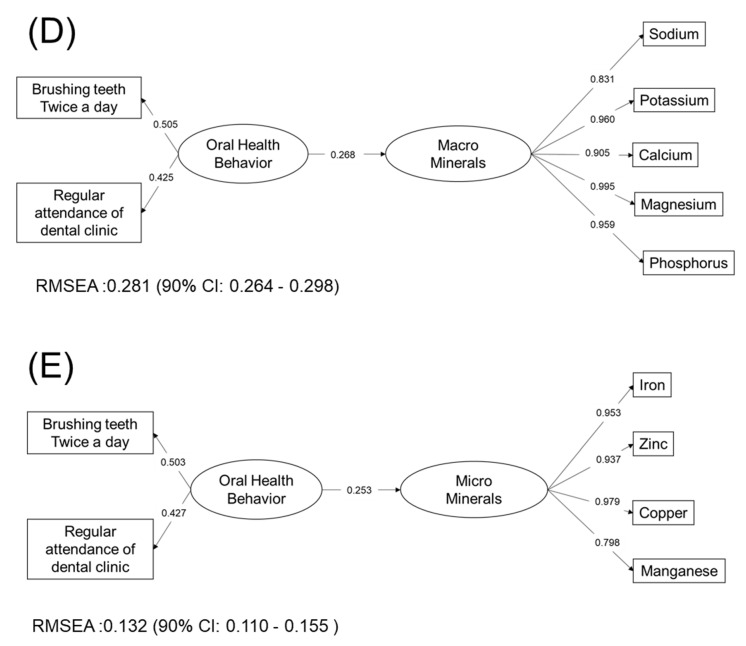
Structural equation modeling of oral health behavior and nutrition intakes: (**A**) three macronutrients; (**B**) water-soluble Vitamins; (**C**) fat-soluble vitamins; (**D**) macro minerals; and (**E**) micro minerals.

Oral frailty screening questionnaire consisted of eight items. The cross tabulations of response of these items and satisfied level nutrients are shown in Table S3. Additionally, the statistical significance of the net value of intakes of nutrients are shown in Table S4. As shown Table S3, oral frailty risk by screening of oral health questionnaire was statistically significant for 10 nutrients by χ^2^ tests. For each item of the questionnaire, brushing teeth twice a day had significant correlations with 20 nutrients and having family dentist had significant correlation with 23 nutrients. In contrast, other items of the questionnaire had correlation with no or only one nutrient (Table S4). A similar tendency could be observed when using the net value of the intakes of nutrients, as shown in Table S4. The number of remaining teeth was only statistically significant for fiber. The results of Structured Equation Modeling (SEM) are shown in Figure 3. All paths were statistically significant except for the path oral behavior to brushing teeth twice a day in the three major nutrients. Paths from oral behavior to nutrients intakes and oral behavior to brushing teeth twice a day and having regular dentist had similar values for water-soluble vitamins, fat-soluble vitamins, macro minerals, and micro minerals. In the model for three major nutrients, the path form oral health behavior to nutrients intakes were smaller than those in other nutrients. The effects of oral behavior on nutrition intakes were different between three major nutrients and other nutrients.

All paths were statistically significant except for the path oral behavior to brushing teeth twice a day in (Figure 3a). Paths from oral behavior to nutrients intakes and oral behavior to brushing teeth twice a day and having regular dentist have similar values in Figure 3b–e. In the model for three macronutrients, the path form oral health behavior to nutrients intakes was smaller than those in Figure 3b–e. The effects of oral behavior to nutrition intakes were different between three macronutrients and other nutrients.

Additionally, the energy balance of three macronutrients regulated by DG were analyzed. The results of cross tabulation by oral health behavior and energy balance of three major nutrients are shown in Table 3. By chi-square tests, energy balance of proteins and fats had a statistically significant correlation with brushing teeth twice a day. However, having a family dentist had no correlation with the energy balance of the three macronutrients.

## 4. Discussion

In this study, we performed a community-based nutritional survey and investigated the relationship among the nutritional intakes, self-assessed oral function, oral health behavior, and number of teeth. The representativeness of study population was compared with the national survey of Japan. Energy intakes of fats and carbohydrates in men were lower than those of the national survey. It indicated that this study population consisted of healthier men.

Consistent nutrition guidelines are essential to improve health. By using the reference values proposed by Dietary Reference Intakes for Japanese [20,21], nutritional status could be clearly categorized for deficiency. Subjects investigated in this study were independent subjects more than 50 years old. Some of the nutrient intakes were not sufficient. Around 90% of subjects had excess salts.

A previous community-based study carried out in Hungary concluded that, for vitamins, the intakes of vitamin B_1_, B_2_, B_6_, B_12_, niacin, and vitamin C are in line with the recommendations [28]. Vitamin D and folic acid intakes are critically low, particularly in the elderly. In this study, 97.9% of subjects’ intake of vitamin B_12_ for EAR was sufficient, while 71.0% for vitamin B_2_ and 62.2% for vitamin B_6_. However, only 35.5% of subjects consumed sufficient vitamin B_1_ for EAR. In contrast, 91.6% of subjects’ intakes of vitamin D for AI were sufficient. Japanese food often uses fish as main dish. These foods are rich in vitamin D. For macro minerals, another study showed that the intakes of iron, copper, and manganese compared with the recommendations were insufficient [29]. In this study, the percentages of subjects with sufficient intakes of iron and copper for ERA were 80% and 86.4%, respectively. In contrast, for manganese, the percentage of sufficient subjects’ intakes was only 40.2%.

The oral frailty screening questionnaire consisted of eight items. Items concerned self-assessed oral functions, daily activity, and oral health behavior. The items concerning self-assessed oral function and daily activity showed almost no significant correlation with nutrition intakes. In addition, the number of remaining teeth had almost no significant correlation with nutrition intakes. In contrast, two items concerning oral health behavior, namely brushing teeth twice a day and regular attendance of dental clinic, had significant correlation with intakes of most of the nutrients. In this study, nutrients intake status was correlated with oral health behavior but not oral functions and number of remaining teeth.

Several studies have shown that oral functions and number of remaining teeth have statistically significant correlation with the mortality of older adults [10]. However, the number of remaining teeth was conflicting. In contrast, oral functions, especially masticatory function, can be the predicator for mortality. A study has shown that oral function and serum levels albumin, which represent the nutritional status, affect mortality independently [10]. In this study, nutritional status had correlation with oral health behavior, which reflects health literacy. 

By the structural equation modeling shown in Figure 3, the coefficients of brushing twice a day were larger than those of regular attendance of dental clinic, indicating that the nutritional intake was dependent on daily health behavior rather than attendance of dental clinic. As primary healthcare, dental clinic has an important role in health promotion. Dental clinic provides treatment with holistic, contentious stance. It should lead to behavior modification [30].

Self-assessed oral function and number of remaining teeth had almost no correlation with the nutritional intakes. Masticatory functions contribute to food digestion and it expands the food choice. Therefore, it can lead to the variation of food and in turn to the improvement of nutritional status. However, food preference or food choice may be robust, and they may not easily change. Therefore, nutritional instruction after oral rehabilitation is necessary. It also plays an important role in the primary healthcare of dentistry.

There are several limitations of this study. The study design is cross sectional. A longitudinal study is necessary to investigate the changes of health status. Information on oral status was limited to the number of remaining teeth. Other oral conditions such as dental caries and periodontal status should be investigated. The oral functions and nutritional intakes investigated in this study were self-assessed. Measuring devices for oral function are available. More precise and objective data on oral function are necessary. Health status other than BMI should be measured. However, overcoming these limitations requires time and resources. This study applied simplified methods to collect larger sample size for the robustness of the data. Propagation of oral frailty has just started in Japan. This study is the first that investigated oral frailty and nutritional intakes. In Japan, the government presents optimal nutritional values by gender and age groups. It is important to evaluate the nutritional intakes of the optimal values by gender, age, and race.

Recovering oral functions by prosthodontic treatment cannot change the nutritional status. In addition to prosthodontic treatment, nutritional instructions are effective to improve nutritional status [31]. Dental clinic is a suitable place for the nutritional instructions along with recovering and maintaining oral functions [32]. 

In conclusion, comprehensive health instructions including nutrition and oral health education are necessary for health promotion. 

## Figures and Tables

**Table 1:** BMI and three macronutrients intakes.

			BMI						
			Categorical Variable				Continuous Variable		
Nutrients	Cut Off	*n*	<Target	Target Range	Target<	*p*-Value	Mean SD	Median 25th–75th	*p*-Value
Proteins (%)	<DG	85	20	37	28	0.017	23.5 ± 3.4 *	23.4 (20.8–25.7)	0.032
	DG	494	147	249	98		22.7 ± 3.1	22.5 (20.5–24.5)	
	DG<	122	35	70	17		24.2 ± 22.4 *	23.2 (20.8–23.6)	
Fats (%)	<DG	58	14	24	20	0.091	23.5 ± 3.3	23.7 (20.5–25.6)	0.062
	DG	385	113	196	76		22.6 ± 3.1	22.6 (20.6–24.5)	
	DG<	258	75	136	47		22.6 ± 3.2	22.4 (20.6–24.1)	
Carbohydrates (%)	<DG	341	75	187	79	0.002	23.1 ± 3.1 **	22.9 (21.1–24.7)	0.003
	DG	336	116	160	60		22.3 ± 3.2 **	22.2 (20.3–24.1)	
	DG<	24	11	9	4		22.2 ± 3.2	21.7 (20.1–24.0)	

*p* values were calculated by χ^2^ tests and Kruskal–Wallis tests. Target BMI ranges are 20.0–24.9 (kg/m^2^) for 50–69 years old and 21.5–24.9 (kg/m^2^) for 70 years or older. Target BMI range is common to men and women. * and ** statistically significant difference by multiple comparison of Dann–Bonferroni method, * *p* = 0.036, ** *p* = 0.002. DG—Tentative Dietary Goal for preventing lifestyle related diseases.

**Table 2:** Frequency of the items of oral frailly scorning questionnaire.

	No		Yes		Missing	
Item of Oral Frailly Scorning Questionnaire	N	%	N	%	N	%
Difficult to eat hard food	572	81.6	127	18.1	2	0.3
Choking	595	84.9	102	14.6	4	0.6
Using denture	371	52.9	323	46.1	7	1.0
Xerostomia	528	75.3	171	24.4	2	0.3
Less frequently going out	571	81.5	127	18.1	3	0.4
Feasible to chew hard food	603	86.0	96	13.7	2	0.3
Brushing teeth at least twice a day	542	77.3	157	22.4	2	0.3
Regular attendance of dental clinic	513	73.2	183	26.1	5	0.7

**Table 3:** Oral health behavior and three macronutrients meets.

		Brushing Teeth at Least Twice a Day			Regular Attendance of Dental Clinic		
		No	Yes	*p*-Value	No	Yes	*p*-Value
Proteins	<DG	31	54	0.001	25	60	0.778
	DG	109	383		127	362	
	DG<	17	105		31	91	
Fats	<DG	21	36	0.006	21	36	0.153
	DG	90	294		99	282	
	DG<	46	212		63	195	
Carbohydrates	<DG	74	267	0.419	86	254	0.085
	DG	75	259		86	246	
	DG<	8	16		11	13	

*p*-values were calculated by Chi-square tests.

## Supplementary Materials

The following are available online at https://www.mdpi.com/2072-6643/12/9/2886/s1, Figure S1: Scatter plot of BMI against proportion of energy by three major nutrients. Figure S2: Number of subjects against sum of sufficient nutrition. Table S1: Comparison with Japanese national health and nutritional survey. Table S2: Difference of number of remaining teeth by BMI and three macronutrients. Table S3: Cross tabulation by nutrition intakes against items of oral frail questionnaire. Table S4: Mean and median values of nutrition intakes against items of oral frail questionnaire.
