# Supplementary material for: Nutritional Status and Oral Frailty: A Community Based Study
Source: Nutrients. 2020 Sep 21;12(9):2886. doi: 10.3390/nu12092886 (PMC7551233; doi:10.3390/nu12092886)
Supplement: Supplementary file 1 [file nutrients-12-02886-s001.zip › Supplementary Materials/Supplementary Materials (figures).docx]

Supplementary materials


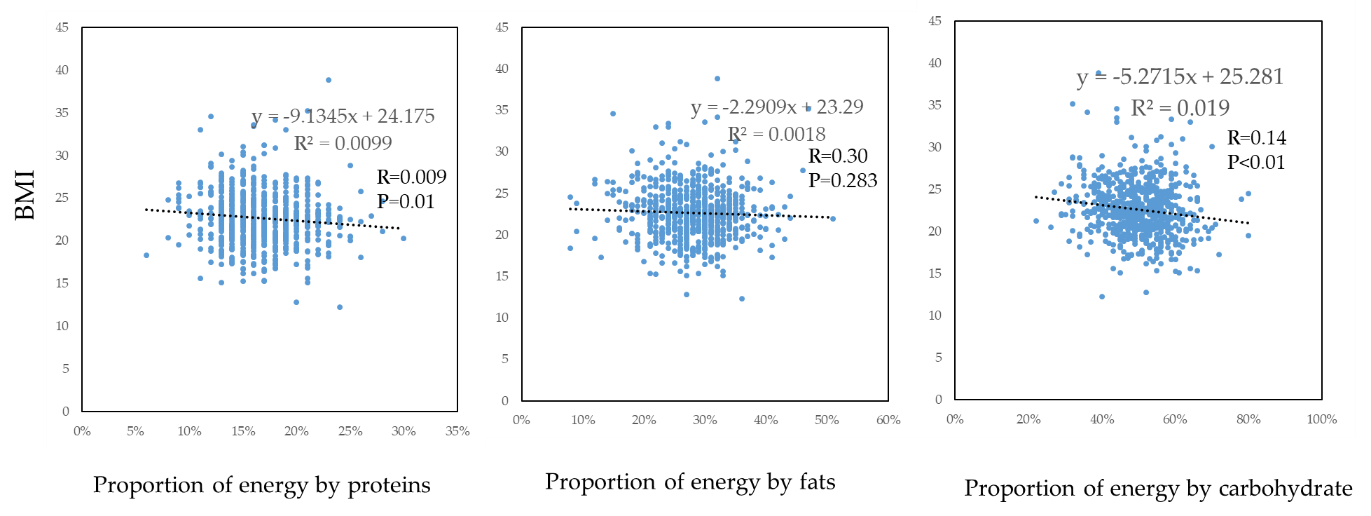


**Figure S1.** Scatter plot of BMI against proportion of energy by three macro nutrients.


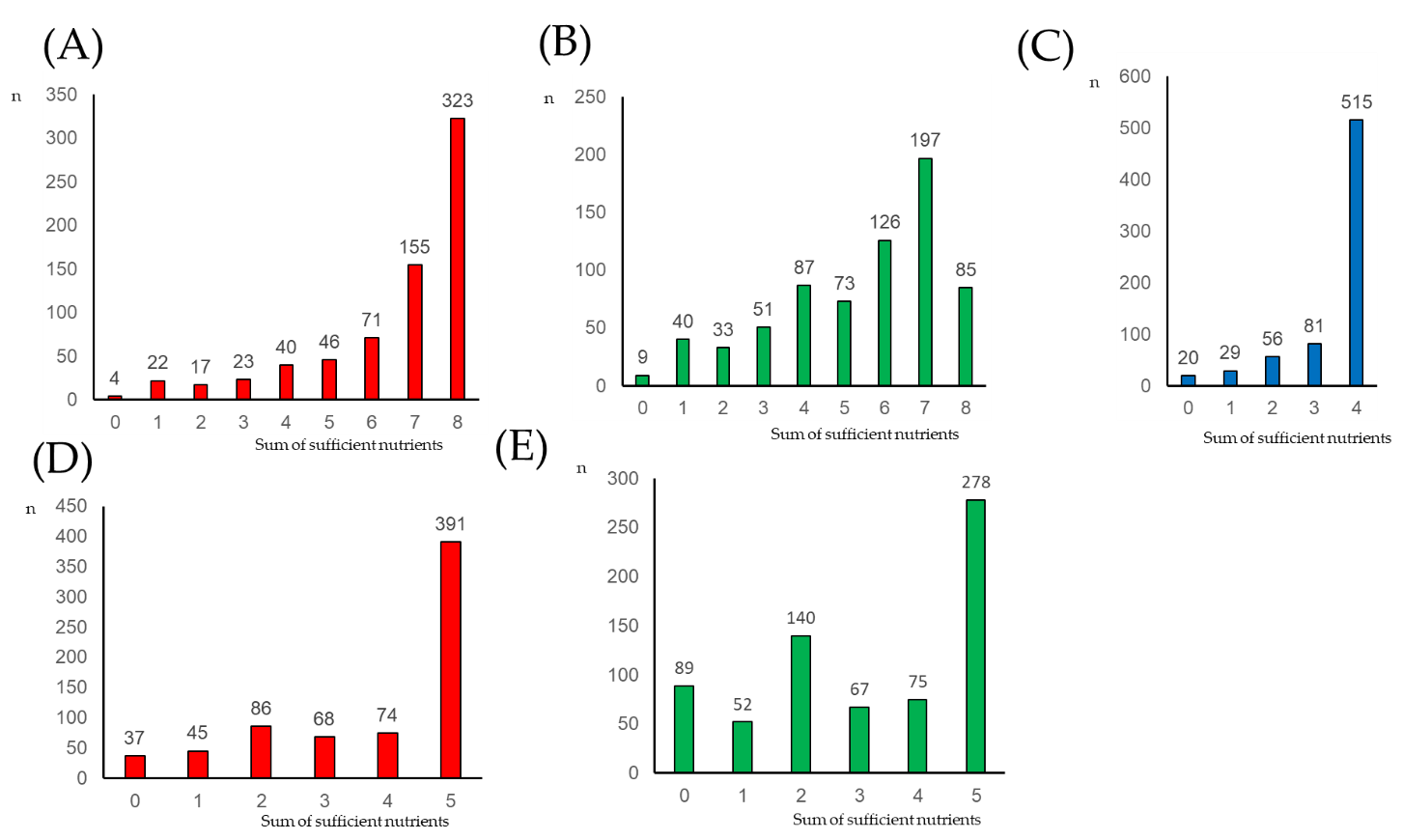


**Figure S2.** Number of subjects against sum of sufficient nutrition. (**a**) Vitamins for EAR, (**b**) Vitamins for RDA, (**c**) Vitamins for AI, (**d**)Minerals for EAR, (**e**)Minerals for RDA. Among the 701 subjects investigated in this study, only 323 (46.1%) satisfied all vitamins EAR levels of vitamins, and 85(12.1%) satisfied RDA levels. For Minerals, 391(55.6%) for EAR level and 278 (39.6%) for RDA were satisfied. ERA: Estimated Average Requirement, AI: Recommended Dietary Allowance, RDA: Adequate Intake, according to Dietary Reference Intakes for Japanese (2015).
